# Role of indocyanine green fluorescence angiography in intestinal obstruction with uncertain ischemia: a case report

**DOI:** 10.1097/RC9.0000000000000805

**Published:** 2026-08-10

**Authors:** Agostina Puente, Raúl E. Croceri, Pablo J. Medina, Matias Mihura, Daniel E. Pirchi

**Affiliations:** Department of General Surgery, Hospital Británico de Buenos Aires, Buenos Aires, Argentina

**Keywords:** fluorescence, indocyanine green, intestinal obstruction, laparoscopy, intestinal viability

## Abstract

**Introduction:**

Intestinal obstruction is a common surgical emergency associated with significant morbidity and mortality, particularly when ischemic compromise is present. Indocyanine green fluorescence angiography (ICG-FA) has emerged as a promising tool for the objective assessment of intestinal perfusion, optimizing intraoperative resection decisions.

**Case presentation:**

An 84-year-old male patient presented with intestinal obstruction secondary to adhesions following conventional cholecystectomy. Exploratory laparoscopy revealed an edematous and congested ileal loop with macroscopically uncertain perfusion. ICG-FA demonstrated homogeneous perfusion compatible with tissue viability, thereby avoiding unnecessary resection. The patient had a favorable outcome and was discharged home on postoperative day 2 without complications.

**Discussion:**

ICG-FA provides an objective assessment of intestinal perfusion. Current evidence suggests that fluorescence patterns can predict tissue viability, overcoming the limitations of classical methods based on direct visualization of bowel loops.

**Conclusion:**

ICG-FA is a useful tool to guide conservative decisions in cases of uncertain intestinal viability, potentially reducing unnecessary resections.

## Introduction

Intestinal obstruction is one of the most common surgical emergencies, accounting for approximately 20% of consultations for acute abdomen^[^1^]^. Its incidence is estimated at 1.47 per 100 000 inhabitants, with a predominance among older adults^[^1^]^. The most frequent causes include adhesions, hernias, tumors, volvulus, intussusception, and endometriosis^[^2–4^]^. Mortality can reach 10% in some series. When it progresses to acute mesenteric ischemia, mortality increases dramatically to 60–80%, a rate that has remained stable in recent decades^[^1^]^.

Laparoscopic treatment has demonstrated the benefits of minimally invasive surgery for the acute surgical abdomen: reduced hospital stay, fewer wound complications, and the possibility of performing a complete 360° exploration of the abdominal cavity. However, the complexity of the laparoscopic approach lies in the presence of intestinal distension, which may predispose to iatrogenic injuries during bowel manipulation. Although historically the laparoscopic approach was limited to highly selective cases, a “laparoscopy-first” approach is now feasible and increasingly used^[^5–7^]^.

In cases of intestinal compromise, a conservative strategy that preserves segments with the potential for tissue recovery should be adopted. This strategy requires objective tools to adequately assess perfusion and to precisely define resection margins, given that classical criteria for determining intestinal viability remain imprecise and poorly reproducible^[^8^]^.

HIGHLIGHTS

- Indocyanine green fluorescence angiography (ICG-FA) enables objective intraoperative assessment of intestinal perfusion.
- Homogeneous fluorescence indicates tissue viability and avoids unnecessary resections.
- Clinical judgment alone has a high rate of potentially avoidable resections.
- ICG-FA is useful in adhesive obstruction, volvulus, and incarcerated hernias.
- Specific equipment and a team trained in minimally invasive surgery are required.

This case report has been reported in line with the SCARE checklist^[^9^]^.

## Presentation of case

An 84-year-old male patient had a relevant medical history of arterial hypertension, benign prostatic hyperplasia, recently diagnosed Parkinson’s disease, and a previous conventional cholecystectomy [right subcostal (Kocher) incision].

He presented with a 3-day history of vomiting and failure to pass stool or flatus, with 24 hours of progressive central abdominal pain. During a previous emergency department evaluation, intestinal distension was noted on an abdominal X-ray, and symptomatic treatment was prescribed. He returned 8 hours later because of persistent pain localized to the right hypochondrium and a new episode of bilious vomiting.

**Figure 1.**
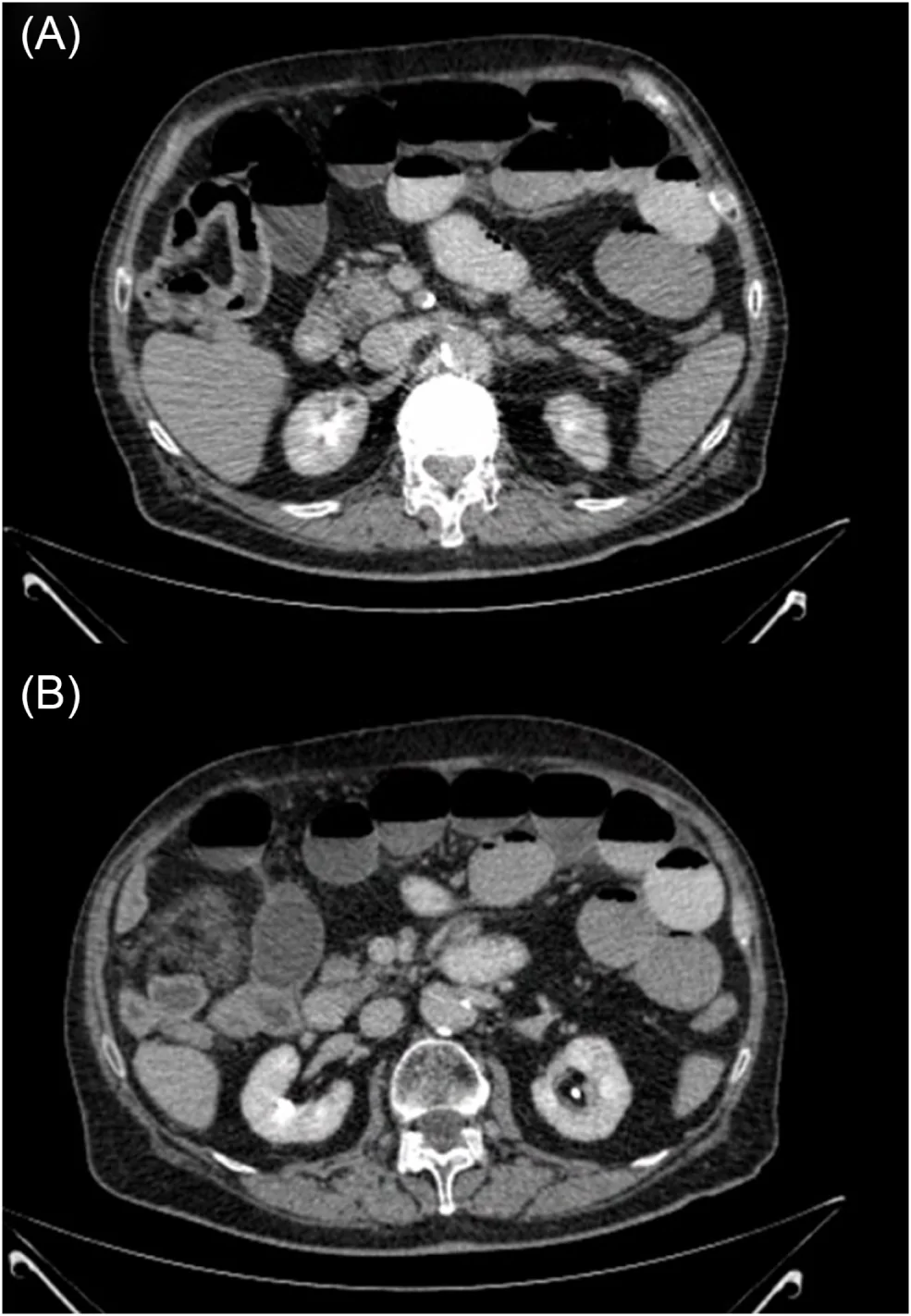
(A and B) Abdominopelvic computed tomography with oral and intravenous contrast showing small bowel distension associated with fat stranding and caliber change at the level of the right hypochondrium with proximal distension and diffuse parietal thickening.

Physical examination revealed an afebrile, hemodynamically stable patient (BP 110/60 mmHg; HR 75 beats per minute). The abdomen was soft and depressible, with tenderness on palpation in the right hypochondrium, without guarding or signs of peritoneal irritation. Rectal examination revealed an empty ampulla. Laboratory tests showed mild leukocytosis (11 800/µL). Abdominopelvic computed tomography with oral and intravenous contrast revealed distension of jejunal and ileal loops with a change in caliber in the distal ileum in the right flank, associated with diffuse parietal thickening, vascular engorgement, fat stranding, and a moderate amount of free fluid between the loops and in the pelvis (Fig. 1, a and b).

Exploratory laparoscopy was conducted under general anesthesia with endotracheal intubation. Skin antisepsis was performed with povidone-iodine. The patient was placed in the supine position with the head elevated. Laparoscopic access was obtained through a 10 mm umbilical trocar for optics, with two additional trocars in the left flank (10 mm and 5 mm).

Laparoscopy revealed marked distension of small-bowel loops and a moderate amount of serosanguineous free fluid in the pelvis. A caliber change caused by an adhesion in the right hypochondrium was identified, which had caused rotation of the antimesenteric border of the affected intestinal segment and was related to the previous cholecystectomy site. The adhesion was carefully released with cold scissors.

**Figure 2.**
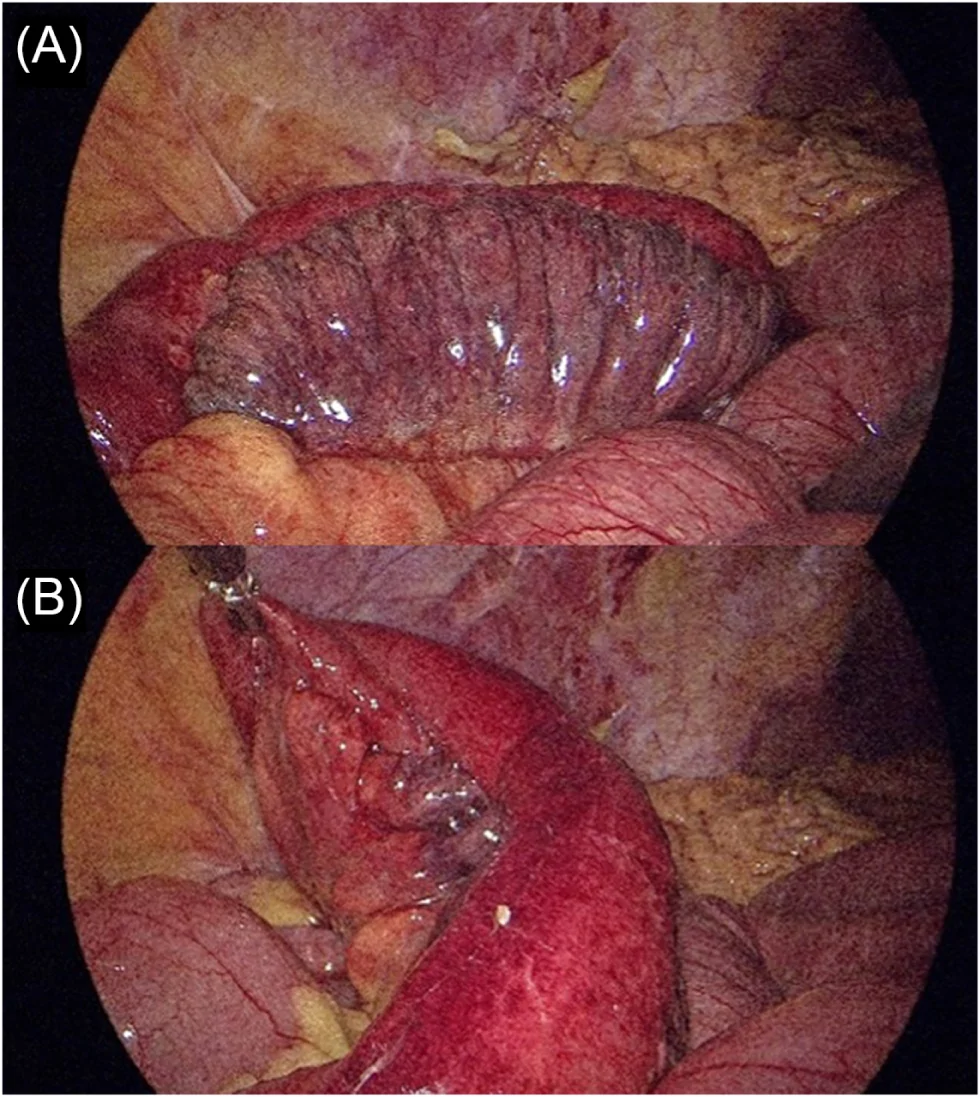
(A and B) Exploratory laparoscopy: edematous, congested distal ileal loop with engorged mesentery.

The abdominal cavity was also explored in a 360**°** fashion, ruling out other sites of intestinal compromise. Systematic running of the small bowel was performed, revealing an edematous, congested distal ileal loop with an engorged mesentery, without areas of frank necrosis but with uncertain perfusion in its most compromised segment (Fig. 2, a and b).

To assess intestinal perfusion, 25 mg of indocyanine green (ICG), diluted in 10 mL of sterile water (concentration, 2.5 mg/mL), was used. Administration was performed via a 5-mL intravenous bolus (equivalent to 12.5 mg of ICG). After injection, fluorescence usually becomes visible within seconds and reaches maximum intensity within 30–60 seconds, allowing dynamic assessment of arterial flow. Fluorescence imaging was performed using a 4K laparoscopic system with integrated near-infrared fluorescence capability (KARL STORZ SE & Co. KG, Tuttlingen, Germany).

**Figure 3.**
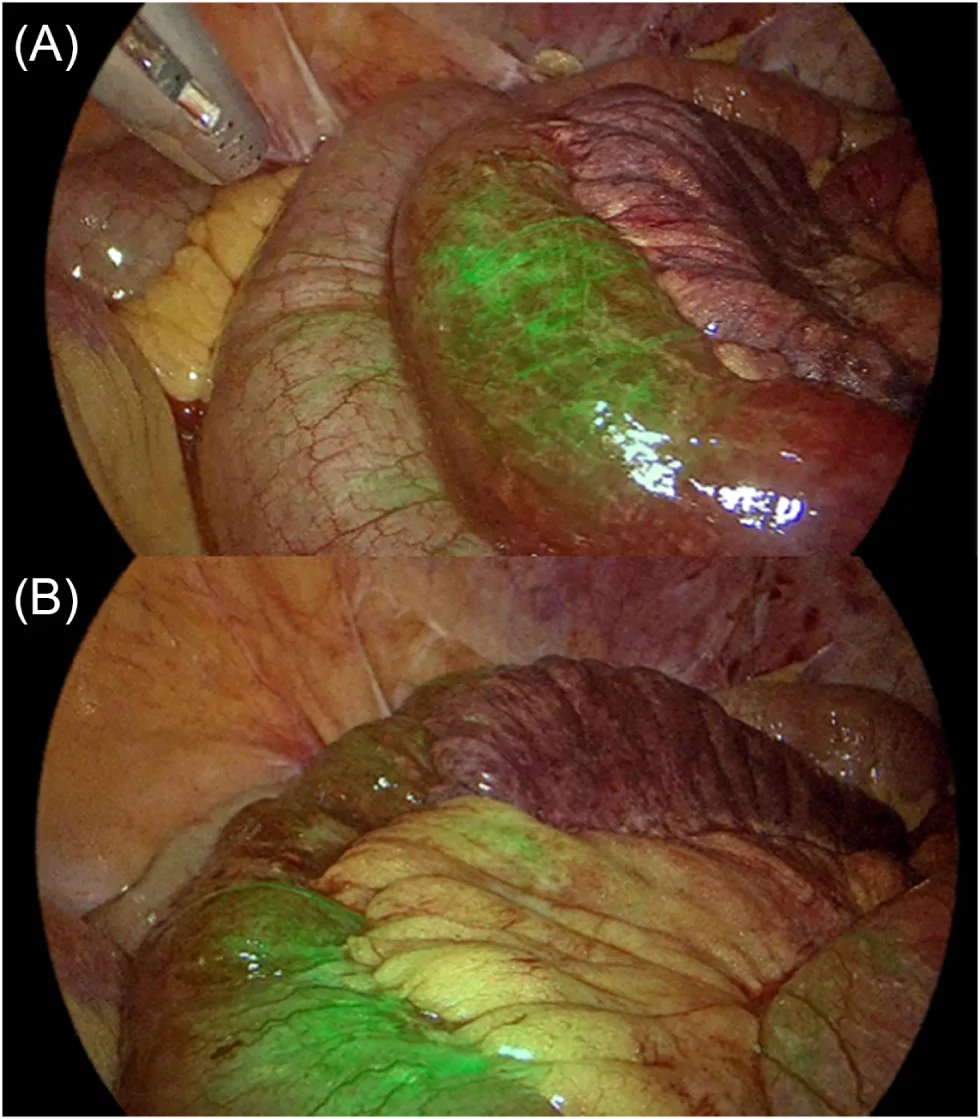
(A and B) Viable intestinal loop visualized by fluorescence angiography.

Fluorescence angiography showed adequate flow in the previously compromised ileal segment, with homogeneous perfusion and no perfusion defects, which allowed us to rule out significant ischemia and avoid unnecessary intestinal resection (Fig. 3, a and b).

The patient had a favorable postoperative course, with adequate oral tolerance at 12 hours post-procedure, progressive recovery of intestinal transit, and no signs of complications. He was discharged 48 hours postoperatively in good general condition.

## Discussion

Intraoperative assessment of intestinal perfusion remains one of the central challenges in the surgical management of intestinal obstruction, especially when ischemia is suspected. Classical methods, including inspection of bowel wall color, observation of peristalsis, palpation of mesenteric arterial pulses, and assessment of marginal bleeding after transection^[^10^]^, have proven to be imprecise and highly dependent on surgeons’ experience, limiting their predictive value in critical situations. This imprecision has direct clinical consequences: studies suggest that standard clinical judgment leads to extensive resections, with reported rates of 39–72.5% for strangulated obstructions, of which up to 46% could be unnecessary^[^11^]^. Furthermore, the macroscopic appearance can be misleading: the mucosa may be ischemic even when the serosa appears intact, which can lead to underestimation of the degree of intestinal damage and increase the risk of subsequent necrosis or anastomotic leak^[^8^]^, as demonstrated by studies showing discordance between macroscopic evaluation and actual perfusion^[^10^]^.

In this context, ICG fluorescence angiography (ICG-FA) offers an objective, reproducible alternative capable of detecting ischemia not evident to the naked eye. Experimental evidence demonstrates that ICG-FA not only confirms the absence of perfusion in clearly ischemic segments but also allows differentiation between reversible damage and lesions with significant capillary alteration, a finding particularly relevant to decisions regarding preservation versus intestinal resection^[^8,12,13^]^.

Recently, Nakashima *et al* published the first cohort study correlating ICG-FA findings with histopathological results in laparoscopic surgery for strangulated intestinal obstruction, proposing a classification of fluorescence patterns that allows prediction of intestinal viability and could standardize decision-making. In cases with a patchy pattern, ICG-FA made it possible to avoid resections that were probably unnecessary ^[^11^]^.

Our case illustrates this scenario: an ileal loop with edema, congestion, and macroscopically uncertain perfusion, in which ICG-FA demonstrated homogeneous fluorescence consistent with viability, thereby avoiding resection. The favorable patient outcome, with discharge after 48 hours without complications, supports the usefulness of this tool in guiding conservative decisions when the clinical evaluation is ambiguous.

Furthermore, integration of this technology into surgical practice appears particularly useful in strangulated obstruction, volvulus, and incarcerated hernias, where intestinal viability is uncertain^[^14^]^. Together, these findings suggest that ICG-FA could become a standard tool for perfusion assessment during emergency surgeries with intestinal compromise, provided adequate equipment is available and sufficient training exists. However, its implementation has limitations: the need for specific equipment (laparoscopic equipment with fluorescence capability), ICG availability in emergency departments, and, fundamentally, a team trained in minimally invasive techniques. Nevertheless, larger series and multicenter validation are needed to consolidate these findings.

## Conclusion

ICG-FA is a useful tool to guide conservative decision-making in cases of uncertain intestinal viability, allowing the avoidance of unnecessary resections. Its implementation in emergency surgery requires specific equipment and adequate training.

## Data Availability

Data sharing is not applicable to this article as no datasets were generated or analysed during the current study. All data supporting the findings of this case report are included within the article.
